# Traits Explain Canopy Tree Occurrence Along Regional Environmental Gradients: A Subset Combine to Be Useful

**DOI:** 10.1002/ece3.73583

**Published:** 2026-04-30

**Authors:** Peter A. Vesk, Saras M. Windecker, Rachael V. Gallagher, William K. Morris, Laura J. Pollock, Isaac R. Towers, Daniel S. Falster

**Affiliations:** ^1^ School of Agriculture, Food and Ecosystem Sciences The University of Melbourne Parkville Victoria Australia; ^2^ Hawkesbury Institute for the Environment Western Sydney University Penrith New South Wales Australia; ^3^ Finnish Museum of Natural History University of Helsinki Helsinki Finland; ^4^ Department of Biology McGill University Montreal Qubec Canada; ^5^ Evolution & Ecology Research Centre University of New South Wales Sydney New South Wales Australia

**Keywords:** eucalypt, joint species distribution models, multi‐level model, trait‐environment associations, trait‐SDM

## Abstract

Trait‐Species Distribution Models (trait‐SDM) help to understand the importance of plant strategies to niches, assess their generality across species and provide a path to predicting species distributions from a shortlist of traits. Yet published trait‐environment associations show considerable inconsistency. Region‐scale models may leverage more species, traits, trait ranges and climatic gradients, than at local scales, while retaining biogeographic coherence, which is lost in global compilations. Here we fit trait‐SDMs with six traits using multilevel models for over 90 eucalypt tree taxa. We model presence‐absence in 1 km^2^ grid cells which contain multiple survey plots, arrayed along environmental gradients which span 120,000 km^2^, 8°C mean temperature and 900 mm annual precipitation. We found stem sapwood density, bark thickness, seed mass and maximum height were the most influential predictors in a multi‐trait model of environmental responses to temperature, water deficit, soil depth and pH. Combined, they explained 9%–19% of variance between species in environmental responses. We found less support for specific leaf area and leaf size. Species occurred unimodally along environmental gradients. Trait‐environment terms indicated species with dense stems were more likely in drier climates, thicker bark in warmer climates and that both thinner bark and larger seeds increased occurrence in shallow soils. Taller species were more common and more likely to occur towards sites that were warmer and wetter than average along the gradient. Our work has wider implications: trait‐SDMs help to test trait‐based theory about realised niches. Single trait models reflect the maximum potential explanation of niche differentiation by a trait, while multi‐trait models represent integrated phenotypes responding to multidimensional niches. For planning and management, such trait‐SDMs can provide useful predictions of *where* certain kinds of species occur or could be restored. But they will leave much uncertainty, especially for identifying *which* particular species occur where.

## Graphical abstract

Our paper addresses the problem of “How to predict where a tree can be found from its traits?” We show that wood density, bark thickness, maximum height and seed mass combine for a useful model of tree distributions.

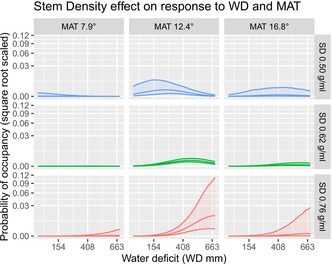

## 1 Introduction

Explaining and predicting species distributions is central to ecology, providing insight to factors that contribute to species' maintaining viable populations and enabling risk assessment for species and spatial prioritisation of management. Species' traits, which represent functioning and fundamental trade‐offs in resource allocation, can help explain *why* species are where they are. In the ideal case, we could take a species for which one just knows a few traits, and predict where the species is likely to be found based only on those traits, and even where it could be found in the future. Despite the intuitive links between species distributions and their traits, in practice, few studies explicitly test this link.

Species distribution models (SDMs) mathematically relate observations of species occurrence or abundance to environmental covariates, reflecting the realised niche (Elith and Leathwick 2009). However, fitting statistical models to occurrence data one species at a time is inefficient, has no predictive capacity beyond the focal species, and leads to little generalised understanding of the mechanisms that enable species to occur where they do. Multispecies (or joint) statistical distribution models (Warton et al. 2015) assume that species share some response to the environment, though also have a species‐specific component to their response (Gelfand et al. 2006; Dorrough et al. 2011; Ovaskainen and Soininen 2011) and have been shown to perform well at predicting occurrence (Norberg et al. 2019). But most SDMs, even multispecies models, do not yet consider traits.

Trait‐environment studies broadly address the relations between traits expressed by organisms and their biophysical environment. Commonly, trait‐environment correlations are estimated from trait values at sites with environmental variables (e.g., Towers et al. 2023) or with community‐weighted‐means regression from vegetation plot data (e.g., Bruelheide et al. 2018). Such correlative or regression analyses of traits on environment are very useful to understand the selection that environment may exert on certain trait values but critically, do not address how traits might affect performance of species under different conditions, nor where *species* occur. Neither can they account for multiple traits. Biophysical (mechanistic) distribution models (e.g., Higgins et al. 2012; Schouten et al. 2020) have the appeal of capturing the mechanisms believed important, but are typically difficult to parameterise for the great diversity of species and environments globally and regionally.

Trait‐Species Distribution Models (trait‐SDMs) combine the species‐based and trait‐based approaches, bridging biophysical explanations or empirical trait‐environment correlations and species locations. Trait‐SDMs build trait‐environment relations into multilevel statistical models of (multiple) species occurrence. Trait‐SDMs enable inference about the association between traits and environment; specifically, the role of traits in explaining species distributions along environmental gradients (Pollock et al. 2012; Jamil et al. 2013; Brown et al. 2014; Ovaskainen et al. 2017; Miller et al. 2019; ter Braak 2019). This feature of traits explaining species distributions offers a general and explicit link between species strategies and performance for sustaining populations under different conditions (McGill et al. 2006; Westoby 2025).

Trait‐SDMs also provide predictions—where species should occur—given their traits and the environment. Potentially this can inform strategies for conservation management and climate adaptation (St‐Laurent et al. 2023). Moreover, one could predict for a given environment and candidate species list, a quantitative ranking of species suitability, which is a growing need for restoration and reforestation actions for biodiversity, carbon and other ecosystem services acting under seed supply constraints (Andres et al. 2024).

Fulfilling the promise of such applications requires trait‐SDMs able to transfer prediction of environmental responses to new species and regions (Vesk et al. 2021). To achieve this, models should be aligned with theory and observations about how species traits influence their responses to the abiotic and biotic environment (Thomas et al. 2019). Identifying which traits to focus on is crucial, as trait data are patchy (Maitner et al. 2023) and published support for strong, general trait‐environment associations is modest (Moles 2018). Considerable inconsistency exists among studies globally with potential context‐dependence at a local scale (Table 1). Region‐scale models may leverage more species, traits, trait ranges and climatic gradients, than at local scales, while retaining global biogeographic coherence (Kambach et al. 2023; Towers et al. 2023; Bouchard et al. 2024).

**TABLE 1:** Counts of trait‐environment associations that were monotonic positive or negative reported in 19 studies (references in File S1).

Trait and theoretical significance	Temperature	Water deficit	Depth of soil	Soil pH
Specific leaf area reflects the leaf economic spectrum from costly, long‐lived leaves to cheap, short‐lived leaves, which are favoured in productive environments (Wright et al. 2004)	+4/−4	+0/−6	+2/−0	+0/−2
Maximum Height reflects allocation to height growth, important in productive environments subject to intense light competition (Westoby et al. 2022)	+5/−1	+0/−1	+0/−2	+2/−2
Seed mass reflects allocation to larger seedlings, these may be more tolerant of harsh regeneration environments (Muller‐Landau 2010), or may be larger when competition sets in (Falster et al. 2008).	+3/−2	+1/−1 (−3T:WD)	+1/−1	+3/−2
Wood density increases construction costs against growth in productive environments (Chave et al. 2009).	+2/−0	+5/−0 (+2T:WD)	+0/−1	+3/−1
Bark thickness is investment in defence or storage away from growth, favoured in less productive, more disturbed environments (Rosell 2016).	+1/−1	+2/−1 (+1T:WD)	+0/−1	+0/−2
Leaf size (Area) is important to leaf energy balance with maximum leaf size limited by low boundary layer conductance in hot conditions, incurring transpiration losses, or radiative energy loss and frost damage in cold temperatures (Wright et al. 2017).	+3/−1	+2/−3 (−2T:WD)		+1/−1

*Note:* Cell entries are the number of significant monotonic responses reported in the indicated (+/−) direction, e.g., +0/−6 for Specific Leaf Area and Water deficit indicates no significant negative relationships and 6 significant negative relationships. The numbers of responses reporting interaction of temperature and water deficit is in brackets (T:WD) in the 3rd column. This reflects that (positive) growth responses to increasing temperature may be expected to diminish as water deficits increase (Francis and Currie 2003; Wright et al. 2017). Note, reported here are broad‐sense trait‐environment associations from diverse analytical approaches including traits as predictors or traits as responses, with data points as individual species records or as community means.

**FIGURE 1:**
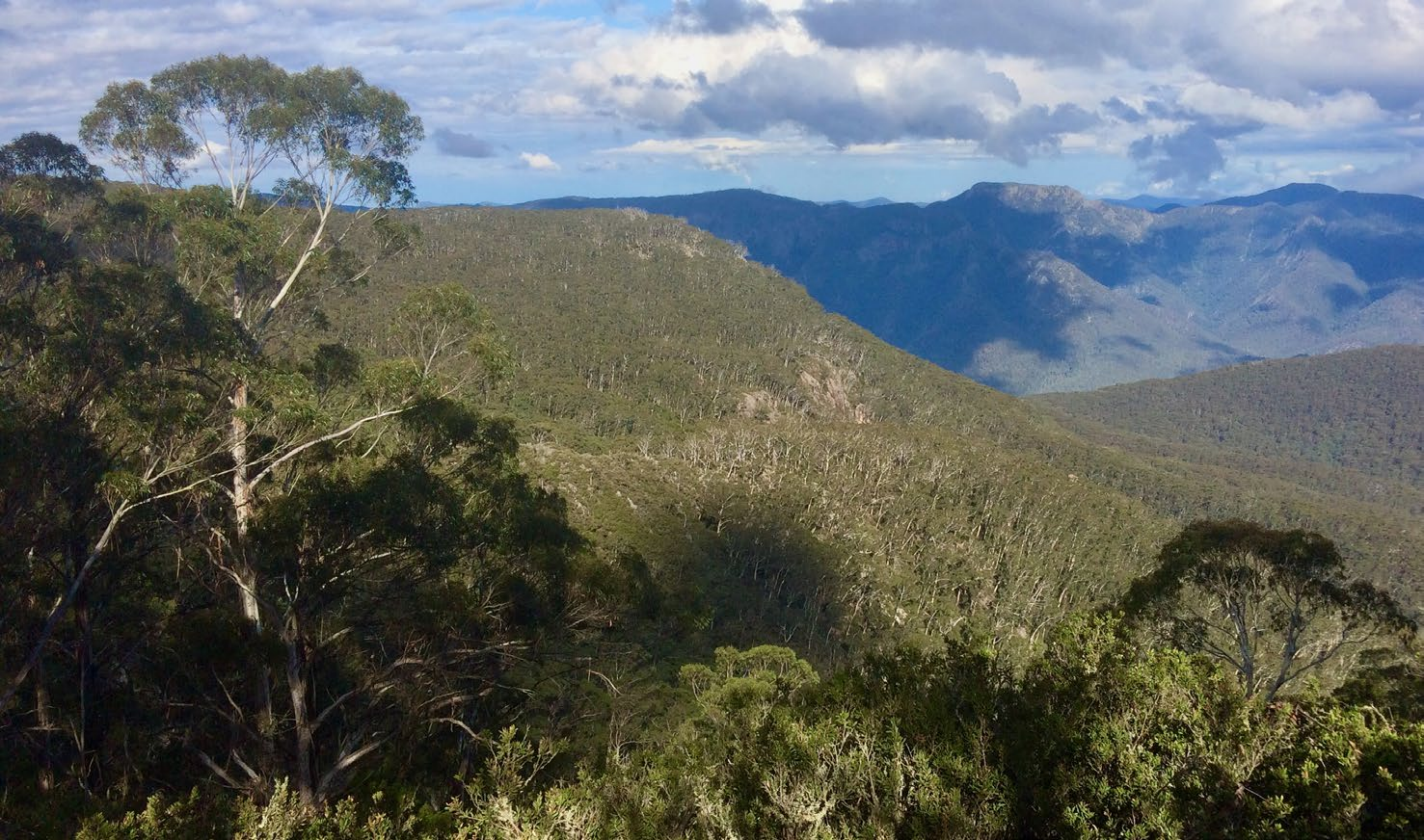
Eucalypt forests dominate landscapes of south‐eastern Australia (photo by Peter Vesk).

Hence, here we work with tractable, parametric trait‐SDM and focus on inference of the trait‐environment parameters among canopy trees at a subcontinental scale. We fit generalised linear mixed models to presence‐absence data on 94 eucalypt trees for 1 km^2^ grid cells across a large region ~120,000 km^2^ supporting forest and woodlands over significant climatic gradients in southeastern Australia (Figure 1). These include some of the most carbon‐dense forests (Keith et al. 2009), some of the tallest forests (Tng et al. 2012), and long environmental gradients with wide variation in vegetation structure (Givnish et al. 2014; Keith 2004).

Our aim is to identify and estimate the trait‐environment terms underlying eucalypt tree distributions and ask how they conform to theory and published empirical findings (outlined in Table 1). We study six traits reflecting aspects of plant strategy: specific leaf area; maximum height; seed mass; leaf size; wood density and bark thickness. We include environmental covariates for climate and soil: temperature; climatic water deficit; soil depth and pH. We include quadratic effects to account for nonlinearity, and interactions between water deficit and temperature. We employ single‐trait and multi‐trait models to understand the role of traits as either independent indicators of strategies or in integrated phenotypes.

Specifically:

1. We estimate how traits are related to the environment by the trait‐environment terms (in sign, magnitude and uncertainty) and use these to predict occurrence across the gradients.
2. We interpret the fitted coefficients and their relation to existing theory and empirical results (Table 1).
3. We assess the relative effects of both climate and edaphic variables in common and in interaction with traits.
4. We ask how incorporating multiple traits informs our understanding of traits' roles in occurrence along multiple gradients by comparison to single trait analyses.

## 2 Methods

### 2.1 Study System and Taxa

**FIGURE 2:**
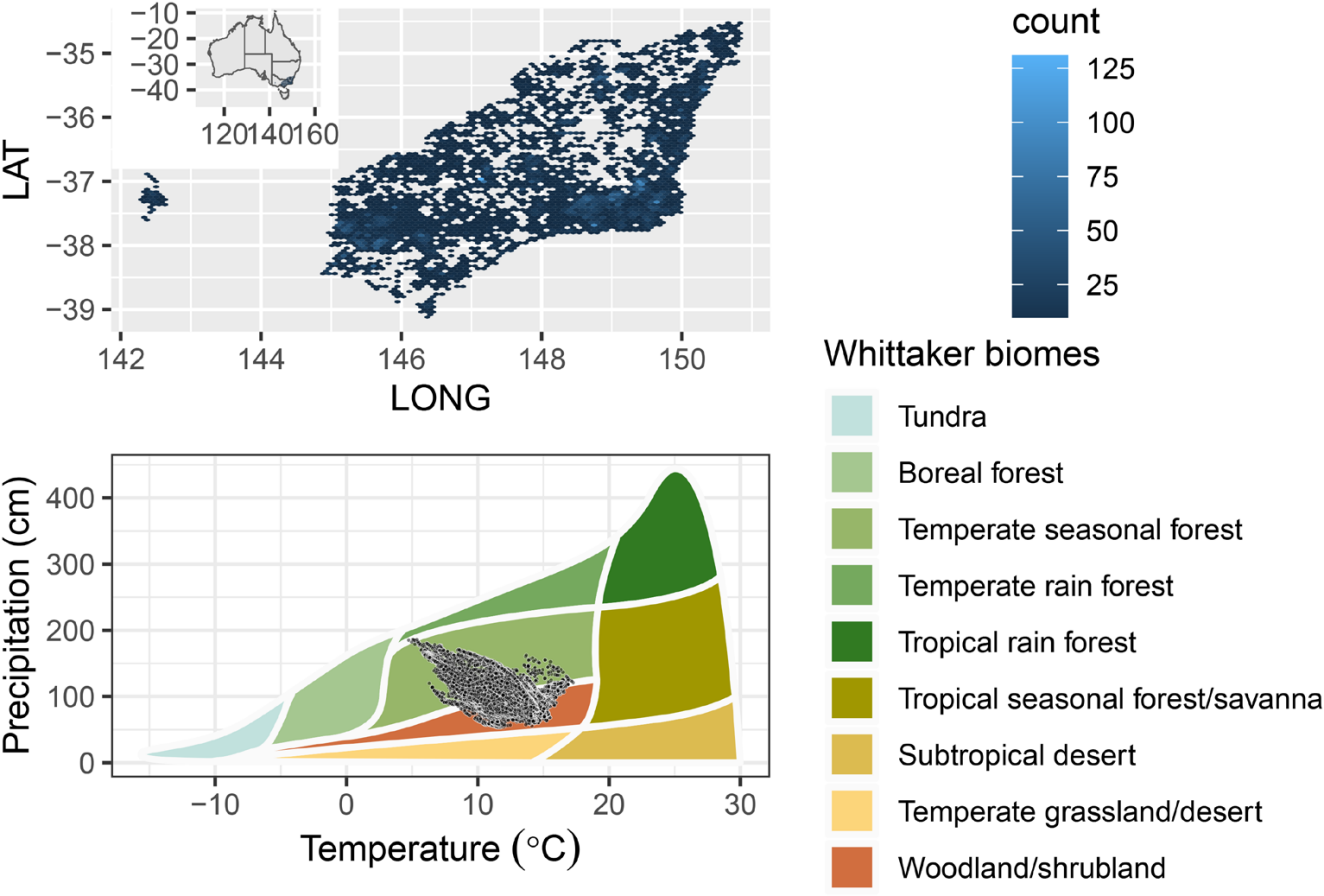
Location and density of surveyed plots within 1 km^2^ grid cells and Whittaker plot of climate.

Our study was conducted in south‐eastern Australia, which has a temperate Köppen climate with mild or warm summers. The extent was roughly 120,000 km^2^, across more than four degrees in both latitude and longitude (Figure 2). The Great Dividing Range dominates the region, and habitats range from coastal plains and valleys through escarpments, low plateaus and mountain ranges, to a maximum elevation of 2274 m asl, and inland slopes. The temperature and precipitation span 7°C–15°C MAT and 700–1590 mm MAP (5th, 95th percentiles). The Whittaker biomes covered are mainly temperate seasonal forest and woodland/shrubland (Figure 2). The vegetation is dominated by eucalypt trees, comprising the closely related genera *Angophora* Cav., *Corymbia* K.D. Hill & L.A.S. Johnson and *Eucalyptus* L'Hér. This clade contains ~900 species that have radiated from rainforest origins into practically all woody vegetation types across Australia. Throughout the study region other woody vegetation types dominated by other taxa (e.g., cool temperate rainforest, sclerophyll heath, swamp thickets) occur typically in small patches < 5 ha. Within the extensive forests and woodlands few non‐eucalypt species contribute significantly to the canopy. Accordingly, and because they serve as a model clade, we focussed on the eucalypts and its close allies in Myrtaceae.

Plot data were compiled from the Victorian Biodiversity Atlas (The State of Victoria, Department of Environment, Land, Water and Planning 2018) and the Southeast forests datasets (Austin and Meyers 1996). These were fixed area plots, 200–2500 m^2^ (90% of which were 900–1000 m^2^) with the presence/absence of all woody species recorded. We considered eucalypt trees (genera *Angophora*, *Corymbia*, *Eucalyptus*) and two close relatives ( *Tristaniopsis laurina* and *Syncarpia glomulifera* subsp. *glomulifera*). Plot data were aggregated to 1 km^2^ grid cells when matching to environmental covariates, such that presence in a grid cell was defined as at least one presence in a plot within that grid cell. Absence in a grid cell was defined by absence from all plots within that grid cell. Subspecies were recognised in 17 cases, aggregates of cryptic species in 10 cases (which may be variably identified in the plot data), and so “taxon” is the correct term, yet we occasionally use the term “species” for simplicity. We excluded taxa that were present in 10 or fewer grid cells, retaining 94 taxa (Appendix A, C: Table A1).

### 2.2 Environmental Data

**FIGURE 3:**
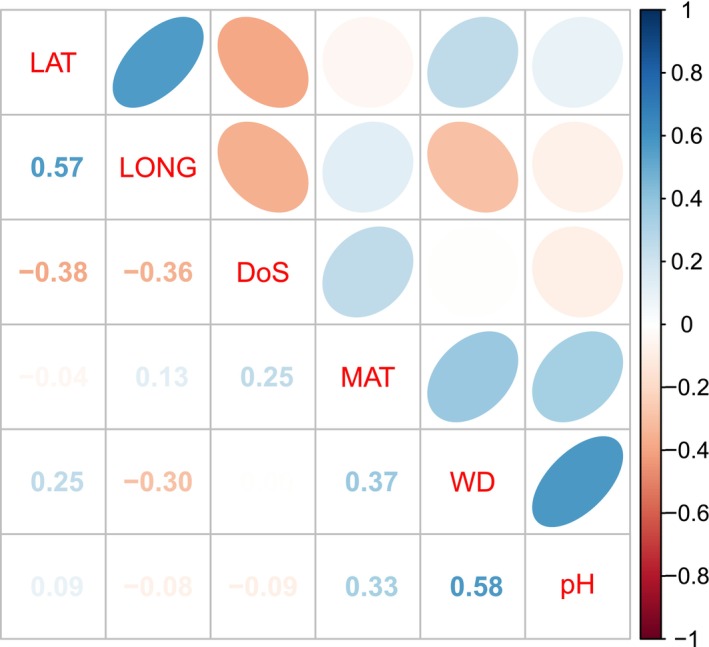
Correlations among environmental covariates.

We used climatic and topo‐edaphic environmental variables available as raster layers. After screening candidate covariates, we chose a subset. Mean annual temperature (MAT; °C) and water deficit (WD; mm annual potential evapotranspiration—actual evapotranspiration) broadly correspond to radiative energy and water available for tree growth. These variables both limit growth and species richness globally (Francis and Currie 2003) and would be expected to have important implications for leaf and stem traits (Wright et al. 2017). We used water deficit (following Francis and Currie 2003) rather than rainfall, as rainfall and temperature were strongly correlated in our data: cold locations are uniformly high in rainfall (*r* = −0.50, Figures 2, 3). Soil relationships to tree occurrence should be modulated by plant traits associated with nutrient and water use, and strategies of resource acquisitiveness versus conservatism (Reich 2014). It is also possible that seed size would modulate species responses to soil gradients reflecting hazards for seedling establishment (Moles et al. 2007) via a tolerance‐fecundity trade‐off (Muller‐Landau 2010). Soil pH (0–5 cm depth) modulates the availability of mineral nutrients and indicates the age and degree of leaching the soil has experienced (Maire et al. 2015). Depth of soil (DoS; m) reflects the accumulation of soil through landscape processes of erosion and transport and indicates the exploitable soil volume for resource acquisition by trees. While soil nutrients and texture would be useful predictors (e.g., using field measured soil texture, see Pollock et al. 2012, 2018), the reliability of GIS layers of soil nutrients is weak (Kooyman et al. 2017). They are based on sparse, biased sampling of soil nutrients and the modelled predictions depend heavily on satellite remote sensing of vegetation, which introduces circularity when attempting to explain variation in vegetation composition and structure. Soil depth and pH are arguably less prone to these limitations. We obtained Mean Annual Temperature (MAT; °C) for 1970–2000 at 30 arcsecond or 1 km resolution from WorldClim v2.1 (Fick and Hijmans 2017) (https://www.worldclim.org/data/worldclim21.html). PET was for 1970–2000 from ENVIREM v1.0 at 30 arcsecond resolution (Title and Bemmels 2018). (https://deepblue.lib.umich.edu/data/concern/data_sets/gt54kn05f). Mean Annual Evapotranspiration (AET) was obtained from the Bureau of Meteorology (https://www.bom.gov.au/jsp/ncc/climate_averages/evapotranspiration/IDCetgrids.jsp) for 1960–1990 at 10 km resolution. Soil covariates were obtained at 30 arcsecond resolution from Searle et al. (2022). Covariates were aggregated to 1 km resolution for modelling. Summary statistics for environmental covariates appear as Table 2, and correlations among them in Figure 3. No correlation between environmental covariates exceeded |0.6|, well below the rule of thumb of *r* < |0.7| so collinearity was not judged to be a problem (Dormann et al. 2013).

**TABLE 2:** Summary statistics of environmental covariates.

Percentile	MAT (°C)	WD (mm year^−1^)	pH	DoS (m)
Minimum	4.74	12	3.88	0.51
25%	10.9	328	4.34	0.87
50%	12.9	407	4.44	0.96
Mean	12.4	408	4.48	0.94
75%	14.1	493	4.57	1.03
Maximum	17.3	770	5.78	1.27

Abbreviations: DoS, depth of soil; MAT, mean annual temperature; WD, water deficit.

### 2.3 Trait Data

We collected all trait data in the field using plot data to guide sampling strategy (except maximum height, see below) (Portelli et al. 2023). Our aim was to maximise the number of species, coverage of trait distributions and habitats in each sampling trip. For practicality, we sampled trees near to roads and tracks within mostly native vegetation, where canopies were accessible with 4 m pole clippers. Occasionally for tall taxa, recently blown‐down branches were used. Traits were measured according to standard protocols (Pérez‐Harguindeguy et al. 2016). These trait data were most recently compiled and analysed in the paper by Portelli et al. (2023), along with methods of measurement. We used traits reflecting leaf (leaf area, LA; specific leaf area, SLA), stem (stem sapwood density, SD; relative bark thickness, RBT), stature (maximum height, MH) and reproductive (seed mass, SM) characteristics (Table 1) known to influence ecological performance (Westoby et al. 2002). Maximum Height was the only trait that we did not measure ourselves; this was sourced from the database Euclid (Slee et al. 2015).

**FIGURE 4:**
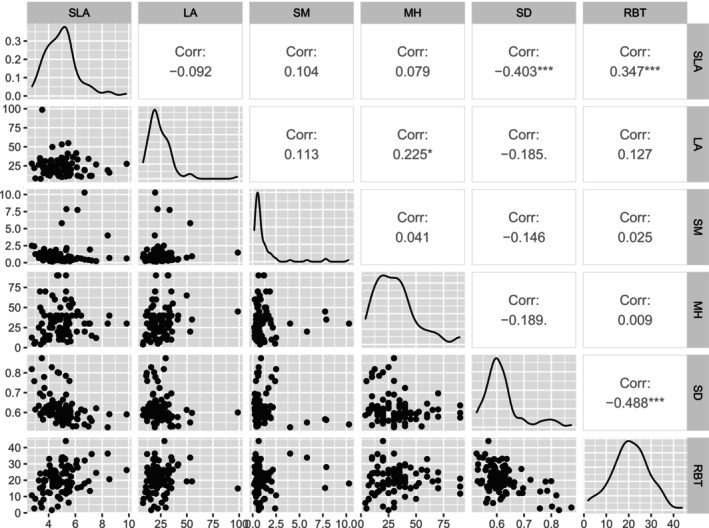
Distributions and correlations among traits in 94 eucalypt tree taxa from southeast Australia, Pearson's correlation coefficients shown. LA, leaf area (8.0–98.7 cm^2^); MH, maximum height (4–90 m); RBT, relative bark thickness (1.7–44 mm at 30 cm girth); SD, stem sapwood density (0.53–0.87 mg mL^−1^); SLA, specific leaf area (2.7–9.8 mm^2^.mg^−1^); SM, seed mass; (0.16–10.3 mg).

We represented taxa with a single trait value, by calculating medians first within, then across, trees within taxa. For aggregate taxa, we finally calculated medians across those taxa. We ignore intraspecific trait variation (ITV) in this instance, though it can be substantial, because our purpose was to build models with species or taxa as the comparative unit. Trait distributions and correlations are found in Figure 4. Stem sapwood density (SD) was the trait most correlated with other traits, being moderately correlated with both relative bark thickness (RBT, *r* = −0.49) and specific leaf area (SLA, *r* = −0.40). SLA was negatively correlated with RBT (*r* = −0.35), together indicating a loose spectrum from trees with dense wood, robust leaves and thin bark, to trees with light wood, thin, flimsy leaves and thick bark. No correlations among traits exceeded |0.6|, well below the rule of thumb of *r* < |0.7|, so collinearity was not a problem (Dormann et al. 2013). To compare and confirm our dataset with other collected values for eucalypt traits, we present quantiles of trait distributions compared with Australian woody plants from AusTraits (Falster et al. 2021) as Appendix B: Table B1.

### 2.4 Statistical Modelling

**FIGURE 5:**
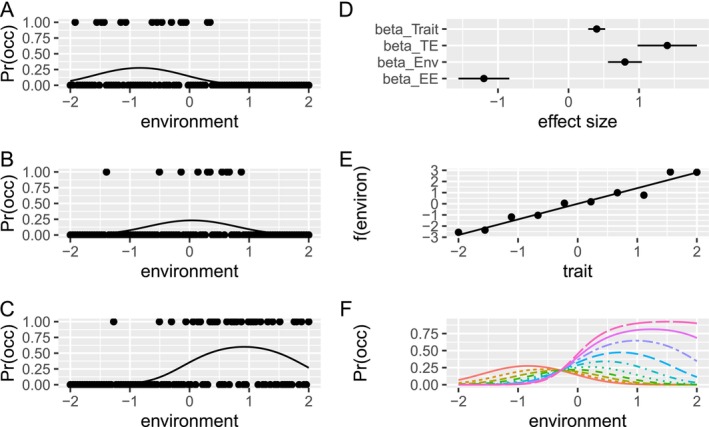
Schematic illustrating trait‐SDM. A‐C show occurrence responses of three hypothetical species to an environmental gradient, with fitted quadratic logistic trendlines. (D) Fitted terms from multispecies trait‐SDM reflecting direct effects of traits (beta_Trait), and linear and quadratic environment effects (beta_Env, beta_EE), and the trait‐environment term (beta_TE), which reflects how the trait modulates the environmental response of species. (E) Displays the trait‐environment relationship (slope of the line = beta_TE), with the hypothetical species‐specific coefficients (dots). (F) Predicted environmental responses for particular trait values.

Our focus was on inference about trait‐environment terms and their impact on predicted distributions along environmental gradients (Figure 5). We built generalized linear mixed models (GLMM) with intercepts and slopes varying by taxon, and fixed effects of environment and for traits modulating those intercepts and slopes. Briefly, occurrence (presence/absence) of the *j*th taxon in the *i*th grid cell, *Y*_*ij*_ = 1 is assumed to be Bernoulli distributed. The corresponding probability, *p*_*ij*_ is modelled as the inverse‐logit of a linear function of taxon‐specific intercepts and coefficients for intercept and covariates that had submodels incorporating the traits (SLA, LA, SM, SD, RBT, MH) and taxon‐level random effects. We fitted independent random effects, as we have no hypotheses for the covariances and wish to maximise statistical power (Matuschek et al. 2017). Here is the equation for a single trait, single environment model with linear effects.

$$\ln\left(p_{\mathrm{ij}}/\left(1-p_{\mathrm{ij}}\right)\right)=\alpha +a_{j}+\beta_{T}T_{j}+\left(\beta_{E}+b_{j}\right)E_{i}+\beta_{\mathrm{TE}}T_{j}E_{i}+\varepsilon_{\mathrm{ij}}$$

$$a_{j}∼N\left(0,\sigma_{a}\right),b_{j}∼N\left(0,\sigma_{b}\right)$$

This may be easily extended to multiple traits and multiple environments and to incorporate quadratic environmental effects.

These may be thought of as multi‐level models with the lowest level the fitted probability of occurrence in a grid cell through parameters for effects of covariates—a standard regression model. Those lower‐level parameters for covariate effects have models themselves with higher‐level (species) parameters determining how species traits and identity influence the environmental responses.

We modelled five environment covariates (MAT, WD, MAT:WD, DoS, pH). Traits were log transformed and then centred and scaled by their standard deviation. Environmental covariates were centred and scaled by their standard deviation. The interaction between MAT and WD was included following previous work that highlighted that leaf‐level energy balance (Wright et al. 2017), productivity, and angiosperm and taxonomic richness (Francis and Currie 2003), may be limited by either water availability or temperature, revealed by their interaction. Biologically, the effects of high temperatures may be exacerbated under water deficit as plants' abilities to cool through transpiration may be impeded. Wright et al. (2017) showed that leaf size was constrained by cold overnight temperatures in wet areas and hot daytime temperatures in dry areas, captured by a statistical interaction between temperature and precipitation. It was not appropriate to fit other (environment‐by‐environment) interactions, as we had no theoretical expectations about other complex environmental gradients. We tested for unimodal responses along environmental gradients with quadratic terms for each environmental covariate.

We measured overall model fit and parsimony with Root Mean Square Error (RMSE) and Akaike Information Criterion (AIC), respectively. We note that these measures focus on the fit of the model to the occurrence data, that is the lowest level of the multi‐level model, rather than on higher level inference about traits on the environmental response, which is our main interest. Selection of traits to retain in more complex models was guided by identifying large, low‐uncertainty trait‐environment terms and by reductions in variance in between‐taxon environmental random effects (indicating that traits explained some of the between‐taxa variation in environmental responses). Variance reduction was estimated for each environmental covariate (and the intercept) and then we used the median across these terms. We used the marginal R^2^ (Nakagawa and Schielzeth 2013) as a measure of the overall variance explained by traits and environment and the Intraclass Correlation Coefficient as a measure of unexplained random effect variance, smaller values being desirable (Nakagawa et al. 2017).

We fitted no‐trait models, single‐trait models, and a series of multi‐trait models all with and without quadratic terms. We broadly proceeded with forward stagewise selection of traits, considering two‐trait models, each with the five environmental covariates. We then considered subsets of three, four and five trait models, guided by between‐taxa variance reduction noted above.

Models were implemented in the R statistical language v4.4.3 (R Core Team 2023) through the R‐studio environment (Posit Team 2024) and models were fit with the package glmmTMB v1.1.10 (Brooks et al. 2017), plots were made using the packages ggplot2 v4.0.1 (Wickham 2016), sjplot v2.8 (Lüdecke 2024) and patchwork v1.3 (Pedersen 2024). Trait correlations were measured with package corrplot v0.95 (Wei and Simko 2024). To aid visualisation, we plotted marginal effects with 90% confidence envelopes, and scaled occurrence axes by square roots. When discussing environmental effects on unimodal models we refer to optimal environments for low or high trait values as ±2 SD of the relevant trait distribution.

## 3 Results

### 3.1 Model Selection

After much preliminary testing with linear models, we report performance of 26 models (Table 3). The model we focus on in the main text (M13) had four traits: stem sapwood density, relative bark thickness and seed mass and maximum height with quadratic effects for all environmental covariates except the MAT:WD interaction. This included the terms with largest effects in single trait models for each of the intercept (MH), temperature (RBT), water deficit (SD), soil depth (SM and RBT) and soil pH (SD). We fitted five models with no traits and found that more complex models were favoured. Quadratic environmental effects were more important to include than the MAT:WD interaction, and varying niche breadths improved fit further, but with severely increased computation time. We compared six single trait models with each of linear and unimodal environmental responses. Uniformly, models with quadratic terms (unimodal environmental responses) had much lower values of RMSE than those with only linear environmental effects (Table 3). We compared nine multi‐trait models, three with linear, and six with unimodal environmental responses. We investigated models with varying niche breadths (quadratic terms varying by species) for two no‐trait and four multi‐trait models, but found them slow to run, with less stability, and we had no hypotheses about what drove niche breadth, so we did not pursue them. We focus on quadratic models in the main text, though both linear and quadratic models are presented in Appendix C Sensitivity Analyses. We note the estimates of linear trait‐environment terms were largely insensitive to including quadratic environmental terms. One exception was for the MAT:WD interaction, which shifted from negative to positive values with the inclusion of quadratic climate effects.

**TABLE 3:** Performance statistics for the fitted trait‐SDMs.

Model	Traits	Env shape	AIC	*R* ^*2*^_*c*_	*R* ^*2*^_*m*_	ICC	RMSE
M0	None	linear	292,659	0.846	0.057	0.837	0.1493
M0.1	None	quad	269,966	0.933	0.197	0.916	0.1446
**M0.2**	**None** ^a^	**quad**	**262,914**	**0.985**	**0.252**	**0.980**	**0.1429**
M0.3	None^a^	quad	265,088	0.983	0.279	0.976	0.1434
M0.4	None	quad	272,800	0.932	0.187	0.916	0.1450
M1	SLA	linear	292,663	0.846	0.076	0.833	0.1493
M2	MH	linear	292,646	0.846	0.128	0.823	0.1493
M3	SM	linear	292,660	0.846	0.065	0.835	0.1493
M4	LA	linear	292,667	0.846	0.062	0.835	0.1493
**M5**	**SD**	**linear**	**292,628**	**0.845**	**0.086**	**0.830**	**0.1493**
M6	BT	linear	292,637	0.843	0.084	0.829	0.1493
**M7**	**SD BT SM**	**linear**	**292,624**	**0.844**	**0.112**	**0.824**	**0.1493**
M8	SD BT SM LA	linear	292,629	0.844	0.116	0.823	0.1493
M9	SD BT SM LA SLA MH	linear	299,205	0.837	0.192	0.798	0.1504
M1.1	SLA	quad	269,969	0.933	0.217	0.914	0.1446
M2.1	MH	quad	269,951	0.932	0.247	0.910	0.1446
M3.1	SM	quad	269,963	0.933	0.207	0.915	0.1446
M4.1	LA	quad	269,973	0.933	0.201	0.916	0.1446
**M5.1**	**SD**	**quad**	**269,941**	**0.932**	**0.222**	**0.912**	**0.1446**
M6.1	BT	quad	269,952	0.931	0.234	0.910	0.1446
M7.1	SD BT SM	quad	269,937	0.932	0.260	0.908	0.1446
M10	SD BT MH LA^a^	quad	272,692	0.926	0.251	0.901	0.1452
M12	SD BT SM MH^a^	quad	292,663	0.846	0.076	0.833	0.1493
**M13**	**SD BT SM MH**	**quad**	**269,926**	**0.932**	**0.304**	**0.902**	**0.1446**
M14	SD BT SM MH^a^	quad	274,375	0.924	0.305	0.890	0.1454
M15	SD BT SM MH^b^	quad	272,759	0.932	0.318	0.900	0.1451

*Note:* Env shape classes the models by whether we included quadratic terms for unimodal environmental responses. The bolded models have the lowest AIC for each class (no traits, linear, quadratic, single‐ trait, multi‐trait. M13 is the selected model in the main results).

Abbreviations: ICC, intraclass correlation coefficient and measures unexplained random effect variance (between species); *R* ^2^_*c*_ the conditional *r* ^2^ and measures variance explained by traits and environment and species identity; *R* ^2^_*m*_, marginal *r* ^2^ and measures the variance explained by traits and environment (but not species identity); RMSE, root mean square error.

^a^ Models included varying niche width.

^b^ Model did not include MAT:WD interaction.

### 3.2 Variation Among Species Responses to Environmental Gradients

**FIGURE 6:**
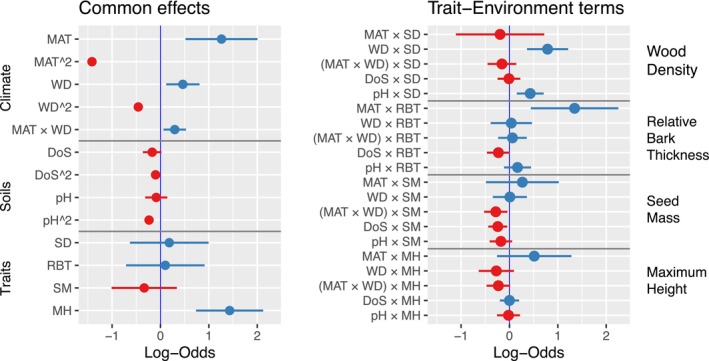
Common effects of Traits and Environment (left) and Trait‐Environment interactions (right) for eucalypt occurrence in the multi‐trait‐SDM. Positive common effects (mean ±90% confidence interval) indicate increase in probability of occurrence of a hypothetical average eucalypt with average traits. Climate variables are Mean Annual Temperature (MAT), Water Deficit (WD). Soil variables are Depth of Soil (DoS) and pH. Traits are Stem Sapwood Density (SD), Relative Bark Thickness (RBT), Seed mass (SM), Maximum Height (MH). Negative quadratic terms (e.g., MAT^2) indicate all environmental responses were unimodal. On average, eucalypts were more likely to occur toward warmer temperatures and at greater water deficits. Eucalypts were more likely to occur in shallow soils. Trait‐environment terms (right) that are further from zero indicate stronger trait influence on a given environmental response. The *x*‐axis represents the effect of a 1 standard deviation change in the indicated covariates, on the log‐odds ratio. In other words, the multiplicative change in the logit probability of occurrence for a one standard deviation change in the covariates indicated.

**FIGURE 7:**
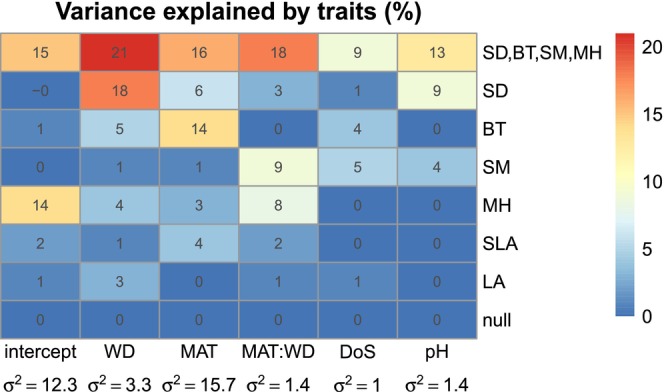
Heatmap of the between‐species variance in environmental responses explained by traits for six single‐trait models and one multi‐trait model. No‐traits model at bottom of figure. Between‐species variance for the no‐traits model is reported in *x*‐axis labels.

Broadly, climatic variables were stronger predictors of the occurrence of an average eucalypt, and had greater between‐taxon variation, than did edaphic covariates (Depth of Soil and Soil pH). Environmental responses were clearly unimodal, as all quadratic terms were highly significant, and clearly negative (Figure 6). Occurrence (and thus species richness) was higher at greater‐than‐average temperature and water deficit. The optimal environmental conditions for the hypothetical average eucalypt were 13.4°C MAT with a WD of 473 mm, 83 cm soil depth and pH 4.4 (Appendix D: Table D1). Variances among taxa in specific environmental responses, which could potentially be explained by species traits, were also larger for climatic than edaphic covariates (Figure 7).

### 3.3 Single Trait Models and Expected Relationships

Traits explaining the most between‐species variation in environmental responses were Stem sapwood density and Bark Thickness (Figure 7). Seed mass and Maximum height were next, with Specific Leaf Area and Leaf Area explaining the least. The overall performance in marginal R^2^ differed in that models of MH and SLA were intermediately ranked owing to explaining variation the intercept (prevalence). In single trait models, species with high SLA were favoured in warm environments (Figure 7, Figure C3). Species with large leaves tended (non‐significantly) to be more likely to occur toward wetter climates (Figure C3).

Regarding the five most consistent expectations for trait‐environment terms from the literature (Table 1) we found: no support for a positive SLA:WD interaction; weak, non‐significant support for a positive MH:MAT interaction; strong support for a positive SD:WD interaction; support for a positive SD:pH interaction and weak, non‐significant support for a negative SM:MAT:WD interaction.

### 3.4 Multi‐Trait Model

The chosen multi‐trait model explained a median of 16% of variance between species across the environmental response terms, compared to a no traits model (Figure 7). Variation in water deficit response was best explained by traits, a 21% drop (from *σ* ^2^ = 3.3 to *σ* ^2^ = 2.6), temperature response was explained similarly 16% (from *σ* ^2^ = 15.7 to *σ* ^2^ = 13.2). Variation in the intercept was explained 15% (*σ* ^2^ = 12.3 to *σ* ^2^ = 10.5). Variation in the MAT:WD interaction was explained ~18% (*σ* ^2^ = 1.37 to *σ* ^2^ = 1.12). Soil responses had slightly lower explanation (pH, from *σ* ^2^ = 1.4 to *σ* ^2^ = 1.2; soil depth response, from *σ* ^2^ = 0.95 to *σ* ^2^ = 0.86).

This multi‐trait model improved explanation of occurrence 10 percentage points for a hypothetical species that was perfectly explained by their traits, over a model with no traits (*R*_m_^2^ = 0.30 cf. 0.20). Yet, species still varied greatly around the expectation based on the four traits indicated by small reduction in large intraclass correlation (ICC = 0.90 cf. 0.92), and persistently high conditional coefficients of determination (*R*_c_^2^ = 0.93 cf. 0.93).

### 3.5 Few Trait‐Environment Terms Were Identified as Important in Multi‐Trait Models

Stem sapwood density and Bark Thickness were highlighted as important traits, through their interactions with environmental covariates of temperature, water deficit and pH and depth of soil (Figure 6). Seed mass had some modest influence through soil depth responses. Maximum height mainly influenced the intercept, with a minor influence on climate response in the multi‐trait model. Most clearly, tall trees were less likely in hot *and* dry climates (a negative 3‐way interaction between MH, MAT and WD, Figure 6) or cool climates.

### 3.6 Stem Sapwood Density and Climate

**FIGURE 8:**
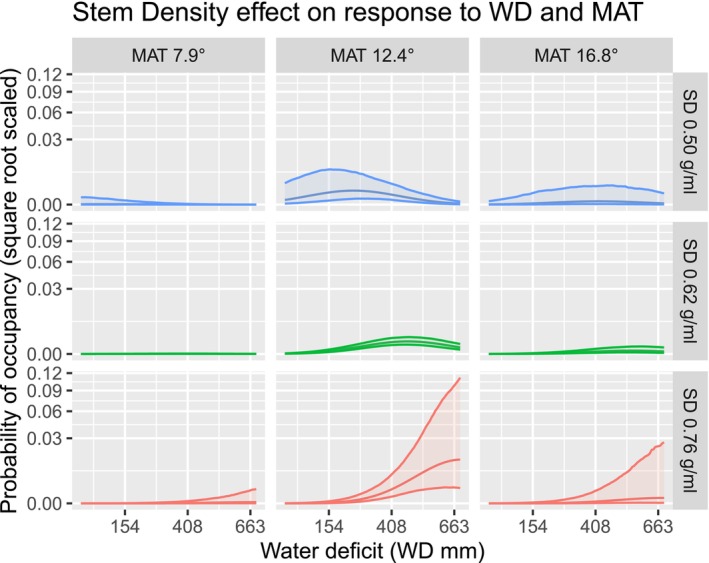
Marginal effects of eucalypt stem sapwood density on response to Water Deficit and Temperature, with all other traits and environment held at their means. Rows display partial responses for taxa with average or high or low (±2 standard deviations) values of the trait stem sapwood density. Columns display conditions of the average or high or low (±2 standard deviations) mean annual temperatures. 90% confidence envelopes. The middle row, being the mean SD is the hypothetical average eucalypt. Y axis is scaled by square roots, but predictions are on the probability scale and not transformed.

Stem sapwood density principally modulated taxon responses to water deficit (Figure 6). Taxa with dense wood (+2 SD) were more likely to occur in dry climates (691 mm optimum) and soft wood taxa were more likely in climates with low water deficits (254 mm) (Figure 8), supporting expectations (Table 1).

The effect of stem sapwood density on differential responses to water deficit was mildly stronger at low temperatures, where only dense wood taxa could tolerate great water deficits. In warmer temperatures, stem sapwood density was less effectual on water deficit response.

### 3.7 Bark Thickness and Climate

**FIGURE 9:**
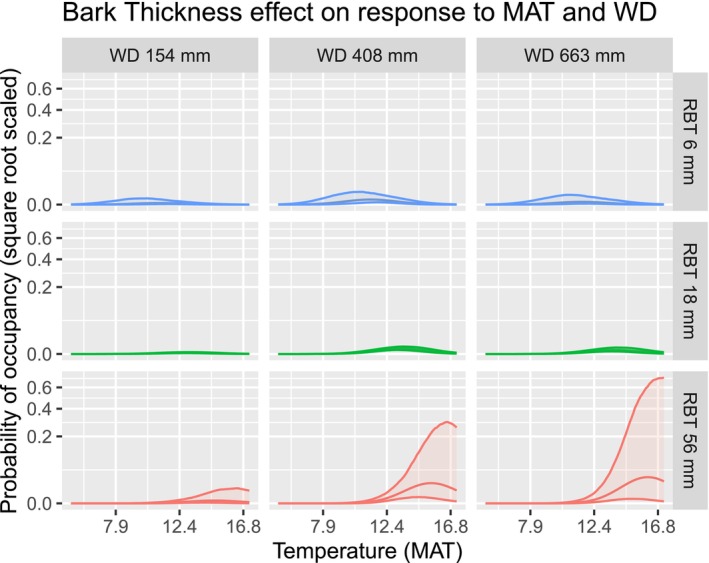
Marginal effects of Bark Thickness on response to Temperature and Water Deficit. Rows display partial responses for taxa with average or high or low (±2 standard deviations) values of the trait bark thickness. Columns display conditions of the average or high or low (±2 standard deviations) mean annual temperatures. 90% confidence envelopes. The middle row, being the mean Bark Thickness, is the hypothetical average eucalypt.

Bark thickness most influenced temperature responses, with thick‐barked species having more positive responses to temperature (Figure 6). This positive response to temperature was increased in drier climates, such that thick bark taxa (+2 SD) were most likely in hot, dry environments (15.5°C, 483 mm) and were much less likely in cool, wet climates (Figure 9). Thin‐barked eucalypts had weaker responses to temperature, tending to be more likely to occur in cooler, wetter climates (11.2°C, 462 mm).

### 3.8 Trait Modulation of Responses to Soil

**FIGURE 10:**
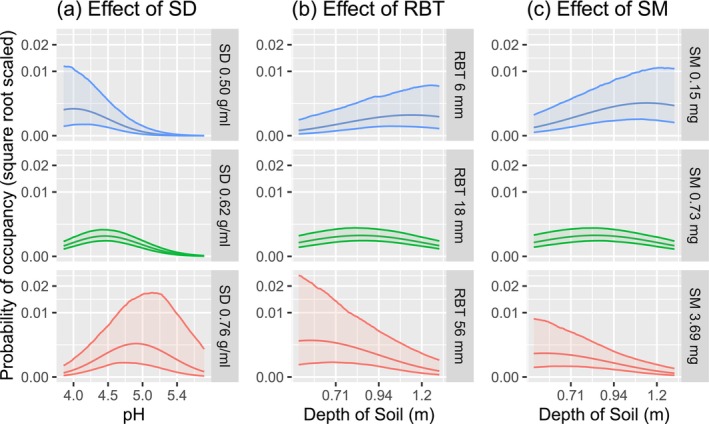
Marginal effects of traits on response to soil variables. Stem sapwood density on response to soil pH (a), Bark Thickness on response to Depth of Soil (b), Seed Mass effects on response to Depth of Soil (c). Rows display partial responses for taxa with average or high or low (±2 standard deviations) values of the trait. 90% confidence envelopes. The middle row, being the mean trait, is the hypothetical average eucalypt.

Trait‐environment terms for soil variables were generally smaller, but more certain than those for climate (Figure 6). Stem sapwood density modulated responses to Soil pH, but not Depth (Figure 6). Taxa with less dense wood (−2 SD) showed a strong negative response to Soil pH, being most common in acidic (pH 4.0) soils (Figure 10). Species with dense wood had a positive response to Soil pH, being more likely in less acidic soils ~pH 4.9.

Bark Thickness had a negative effect on taxon response to Depth of Soil, but no effect on Soil pH response (Figure 6). Thick‐barked species (+2 sd) were more likely to occur on shallower soils (0.58 m) and decline toward deeper soils (Figure 10). Whereas thin‐barked species (−2 SD) were rare on shallow soils and increased weakly toward deeper soils (optimal 1.1 m).

Seed Mass had a negative effect on species' response to Depth of Soil (Figure 6). Heavy seeded taxa (+ 2 SD) were more likely to occur on shallow soils (0.57 m optimum) and to decline towards greater Depth of Soil (Figure 10). Light‐seeded taxa (−2 SD) were more likely toward deeper soils (1.1 m optimum).

## 4 Discussion

The major findings of this work are that wood density, bark thickness, seed mass and maximum height together could explain some 9%–19% variation in occurrence responses to environment of eucalypt trees in SE Australia, through temperature, water deficit, soil depth and pH, with a marginal *R* ^2^ of 0.3. This is in the range of (broad sense) trait‐environment relationships (Moles 2018) and for trait‐SDM specifically (Pollock et al. 2018; Vesk et al. 2021; Laughlin et al. 2023; Rolhauser et al. 2021). Realised niches were clearly unimodal; select traits had directional effects on occurrence along environmental gradients. Single‐trait models indicated more trait–environment relationships, but several were subsumed in multi‐trait models, notably leaf traits. We discuss the trait‐environment terms in greater depth below, with reference to our expectations.

### 4.1 Traits That Mattered in a Multi‐Trait Model

#### 4.1.1 Stem Sapwood Density

Sapwood density represents a growth‐defence trade‐off and potentially a safety‐efficiency trade‐off in water supply (Hacke et al. 2001; Chave et al. 2009; Towers et al. 2024). Our work supports these interpretations. Interestingly, wood density has been variably related to precipitation, potentially because of region‐specific confounding with temperature (Towers et al. 2023). Mechanistic modelling predicts wood density to increase with lower soil moisture, but with less effect from vapour pressure deficit (Towers et al. 2024).

The positive sapwood density‐pH relationship we found supports previous work (Simpson et al. 2016; Bruelheide et al. 2018; Guerin et al. 2022), and deserves deeper mechanistic attention. One idea is that high pH reflects greater clay content and less sand, which influences water availability at the driest times (Maire et al. 2015). Clay soils may exert more negative soil water potentials in dry times than sandy soils, all else being equal (Fernandez‐Illescas et al. 2001). Yet it is also possible that the relationship we found is a spurious correlation through higher rainfall regions having lower pH soils and the greater water availability selecting for lower wood density.

#### 4.1.2 Bark Thickness

Bark thickness has primarily been considered by ecologists to reflect allocation to defence away from growth, and particularly for fire defence (Dantas and Pausas 2013; Jackson et al. 1999; Lawes et al. 2013; Lawes and Neumann 2022). Yet bark is structurally and functionally diverse, in particular, the live, inner bark is important to internal transport and storage of water and non‐structural carbohydrates (Rosell 2019). We found strong evidence that thick barked species are more likely at warmer temperatures, suggesting that warmer climates select more for defence and/or storage and cooler climates for growth. This supports the multi‐continental study of Rosell (2016), but counters Simpson et al. (2016).

Rosell (2016) also found total thickness of bark positively correlated with fire frequency. Whether bark thickness is intrinsically related to climate, or the fire regime that the climate produces, we cannot say. Fine‐grained covariates of fire regime are not available for our region. Yet, broad patterns exist with implications for bark (Murphy et al. 2013; Lawes and Neumann 2022). Low productivity woodlands of multi‐stemmed (mallee) eucalypts in dry or cold climates experience infrequent, medium‐intensity crown fires. These eucalypts have thin bark, as the stems do not grow large enough to survive heating from fire, and resprouting from lignotubers ensures persistence. Grassy woodlands experience infrequent low‐intensity surface fires that individuals can survive from epicormic buds and intermediate bark thickness. Open forests experience frequent, low intensity surface fires and medium intensity crown fires. These often support thick barked species. The productive, tall moist forests experience infrequent, high severity crown fires. These support obligate seeders and epicormic resprouters with thin bark.

We also found bark thickness correlated weakly, negatively to soil depth, indicating thinner bark species on deeper soils (supporting Jager et al. 2015), again potentially indicating selection for growth and away from defence or storage. We urge more work on bark thickness. Both in the sense of data and analyses, but also in fundamental functional biology and theory for its mechanistic importance to species fitness across environments.

#### 4.1.3 Seed Mass

Tolerance‐fecundity theory (Muller‐Landau 2010) suggests larger seeds under harsh conditions, where large seedlings or deep roots are an advantage (Leishman et al. 2000). Our results of big seeds (though small relative to angiosperms broadly) favouring occurrence on shallow (and acid) soils provide modest support for tolerance‐fecundity theory. It also supports some empirical work (Guerin et al. 2022) but contradicted others (Jager et al. 2015).

Towers et al. (2023) found a weak positive relationship of seed mass to annual precipitation (*r* ^2^ = 0.12 for woody species across Australia). So, a lack of climate effects in our study is unsurprising, noting our study's comparatively narrow range of rainfall and seed mass (Appendix B). Studies in Table 1 provided mixed expectations across all gradients, except for small seeds in hot and dry conditions (Bruelheide et al. 2018; Treurnicht et al. 2020), which our results support (Figure 6).

More broadly, the variance among species in coexisting species is large compared with the shift in means along environmental gradients (Moles et al. 2007). Something spreads out the seed size distribution (e.g., game theory), or the fitness consequences of seed mass variation are slight.

#### 4.1.4 Maximum Height

Maximum height in our data varies widely—from 4 to 90 m. So, the effects of maximum height on environmental responses we found seem modest. A striking gradient in heights can be found from nw Victoria to the Central Highlands (Givnish et al. 2014). The tallest forests across our region are found at intermediate elevations ~200–1100 m asl ( *Eucalyptus regnans* ) on deep fertile soils on poleward‐facing slopes sheltered from high irradiance and thus fire risk (Ashton 1981; Mifsud 2002). Givnish et al. (2014) explain the variation across this gradient through a strong gradient in water availability as measured by the ratio of precipitation divided by pan evaporation. Yet, continuing to higher elevations, forest heights decline again until (species‐poor, low abundance) subalpine woodlands at 1500–2100 m asl have low heights (< 15 m) and are occupied by short species like the snowgums ( *Eucalyptus pauciflora* sensu lato, and Black Sallees *Eucalyptus stellulata* ).

So, throughout our region, maximum height is probably limited by two factors—water deficit and cold (frost‐causing) temperatures. These combine to limit maximum height to unimodal peaks along the temperature and water deficit gradients, as our results reveal, though not as obviously as expected. The optimal climates for the tallest trees (mean + 2 SD) were 14.2°C MAT and 397 mm.year^−1^ WD.

The dominant effect of Maximum Height in the multi‐trait model was on the intercept, meaning that taller species are more common. This suggests environments that were best suited for the average tree had greater likelihood of supporting tall, rather than short, trees. This makes sense in that full expression of the tree growth form—tall height—is only achievable under the most suitable conditions for trees. Whether taller species having greater likelihood means they are more competitive than shorter species is beyond these data, though is plausible (Kunstler et al. 2016).

### 4.2 Some Commonly Studied Traits Had Little Effect

We found limited support for SLA and Leaf Area modulating environmental responses. In contrast, the most recent, comprehensive Australia‐wide analysis of traits explained by environment showed strongly increased LA, and SLA towards higher Mean Annual Precipitation (Towers et al. 2023). They found Vapour Pressure Deficit to explain very little, except that SLA (in that study, 1/SLA, *r* ^2^ = 0.10) declined in drier climates.

How to explain that of such commonly studied traits (Table 1), SLA, LA and MH contribute little to explaining variation in species occurrence along gradients? Does this mean that they are unimportant, or that aspects of our study reduce or confound power to detect real effects? Obvious study aspects with potential to limit detection of real effects are the species set, the trait ranges, the environmental covariates chosen, environmental extent and its complexity (Pollock et al. 2018). Possibly disturbances like fire exert a strong effect on species' realised niches, relative to climate and topo‐edaphic variables we studied.

First, this study only concerns a single clade of trees (eucalypts: *Angophora*, *Corymbia*, *Eucalyptus*) and this might constrain the trait ranges and hence limit the power and robustness of our analyses (Wright et al. 2005). While some species are mallees (multi‐stemmed individuals) this is, at most, only two growth forms relative to the wide variety of possible growth forms in the flora. Indeed, much variation in traits like SLA and height occurs across growth forms (Díaz et al. 2016; Wright et al. 2005; Moles et al. 2009) rather than within. Across our > 90 species, SLA ranged 3.6‐fold, narrow relative to the rule of thumb of fivefold variation for leaf trait relationships suggested by Wright et al. (2005). Leaf size ranged 12‐fold, which is substantial (though narrow relative to the AusTraits data set, LA ranges 1560‐fold from 0.02 to 314 cm^2^; 1%–99%) and MH (22‐fold) is very wide (Figure 4, Appendix B). Seed mass had 64‐fold variation while SD varied 1.7‐fold.

Our species set excludes rainforest trees—and their somewhat specific suite of traits—from in restricted areas with damp, deep fertile soils that are largely fire‐free (Bowman 2000). Genus *Acacia*, containing many species, tend not to occupy the canopy. Their inclusion would extend, on average, into faster leaf economics, lower wood density, and larger seeds. Other notable taxa across the region that we exclude are the genus *Callitris* (family Cupressaceae, Gymnospermae) in dry climates on sandy or rocky soils, and family Casuarinaceae, which may dominate some lowland riparian areas and occur as accessory species in dry forests. This limitation of taxa potentially reduces the trait and environment extents available to analyse.

#### 4.2.1 Specific Leaf Area

We found weak support that higher SLA eucalypts were more common in warmer locations, with uncertain effects on water deficit responses. SLA was correlated with stem sapwood density (*r* = −0.45) and Bark Thickness (*r* = 0.37), which both explained much more between‐species variance than SLA. So, while there was a spectrum from thick‐barked trees with high SLA and low stem density to thin‐barked trees with low SLA and dense wood, that spectrum did not display correlated environmental responses. Instead, what correlation SLA had with temperature and water deficit responses was better captured by Bark Thickness and Sapwood Density, respectively. SLA is at the hub of a network of traits across southern Australian eucalypts (Portelli et al. 2023). Having to coordinate with other traits (SD, BT) that are selected along distinct gradients may partly explain why SLA showed weak trait‐environment relationships.

#### 4.2.2 Leaf Size

Wright et al. (2017) showed how, in cold environments, maximum leaf size was limited by frost damage due to radiative heat loss overnight and, in warmer and drier locales, transpirational losses to cool large leaves. A strong relationship between leaf size and climate is expected where only one of these processes act substantially. In our region, it seems both processes act moderately, without either dominating (see Wright et al. 2017), obscuring any clear trait‐environment signal. Potentially also the pendulous leaf habit of eucalypts may help them avoid heating.

### 4.3 Theory and Response Shape

These trait‐SDMs are correlative models of occurrence that reflect the realised niche of eucalypts. These inferences are cross‐species inferences about current patterns, not statements of evolutionary adaptions throughout the eucalypts. Our inferences thus reflect contemporary occurrence, in the presence of dispersal and biotic interactions, notably competition. Broadly, we expect shared preference niches (McGill et al. 2006), with a trade‐off among species of dominance—tolerance (Orians and Solbrig 1977; Smith and Huston 1989) whereby all species do best in the most favourable sites (benign conditions, abundant resources). But the fastest growing species (most profligate in use of resources, or efficient at deploying biomass into new assimilatory organs) grow fastest there and competitively dominate others. Under resource scarcity or otherwise adverse conditions that limit growth, the performance of faster growing species declines greatly, and conservative growth strategists can maintain a positive balance of biomass production and consequently win in competition. The net result of such a dominance—tolerance trade‐off in a competitive setting is a series of unimodal abundance responses arrayed along gradients of favourability. Mechanistic Trait‐Growth theory (Towers et al. 2024) produces such shared preference niches along gradients of water availability rates for single phenotypes. A future direction would be to model community dynamics using such trait‐growth theory models.

**FIGURE C2:**
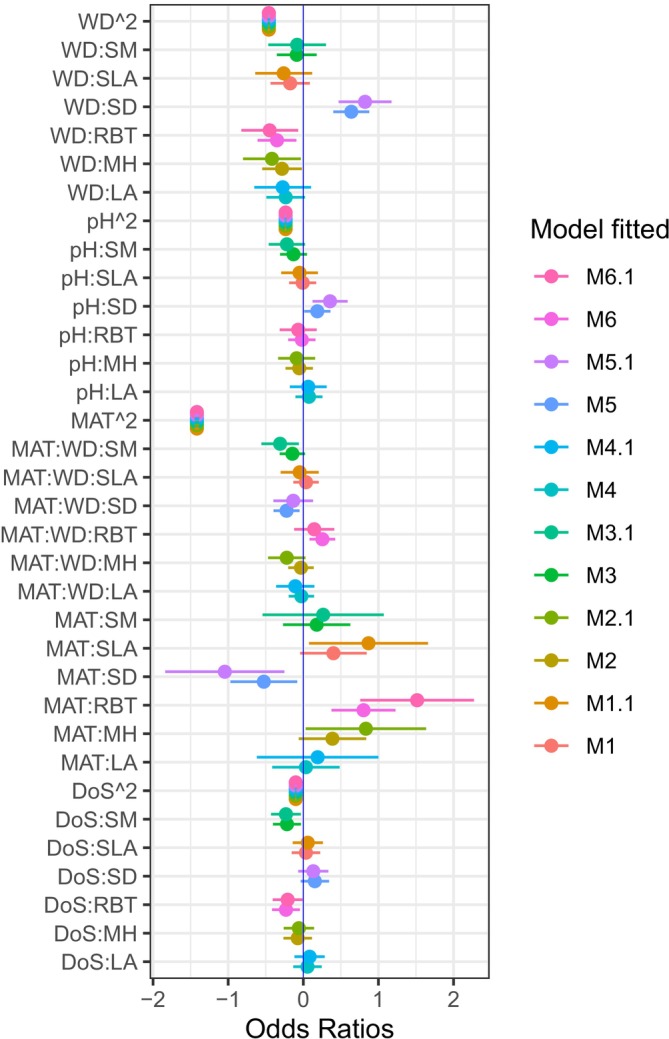
Coefficients and 90% CIs for trait‐environment terms for single trait models with linear (M1–M6) or quadratic responses (M1.1—M6.1) to the environmental gradients. Illustrates that trait‐environment terms inferred from models assuming linear or quadratic responses to environment are similar.

**FIGURE C3:**
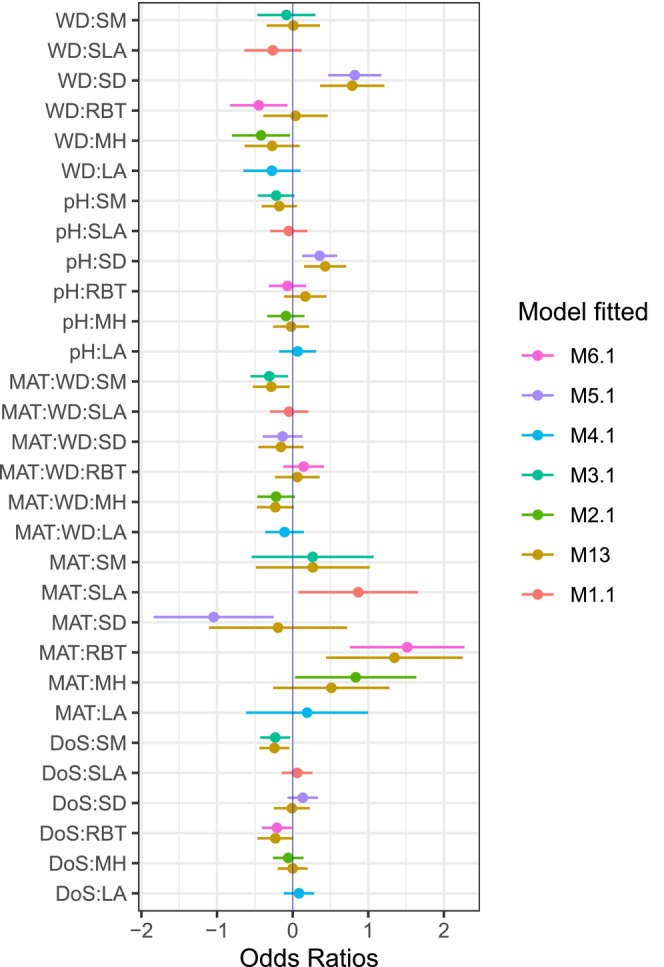
Coefficient plot illustrating trait‐environment terms from 6 single trait models assuming unimodal responses to environment: M1.1 (SLA), M2.1 (MH), M3.1 (SM), M4.1 (LA), M5.1 (SD), M6.1 (RBT) and our selected multi‐trait model M13 (SD, RBT, SM, MH). Symbols are estimated coefficients and 90% CIs as bars.

However, the trait‐environment responses are not so obviously unimodal (ter Braak 2019). Other, similar work has fitted quadratic responses, but with little evidence of widespread, strong, unimodal trait‐environment responses (Rolhauser et al. 2021). We found that fitting quadratic terms improved the fit considerably, by allowing species responses to be unimodal. Yet, trait‐environment terms were little changed when comparing unimodal or linear species responses (Figure C2). This suggests that simpler models assuming linear response to environmental gradients may still provide useful first‐order inferences about how traits influence performance along environments, even though they may not capture individual species responses well.

One effect of specifying quadratic terms for environmental responses is that the direction of the common response to the MAT:WD interaction was reversed, compared to the linear models. When we specified linear response to temperature and water deficit, occurrence increased toward warmer or drier climates, but not to *both* warmer *and* drier (Francis and Currie 2003). This supports the idea that in dry conditions the negative effects of high temperature cannot be offset by transpirational cooling (Wright et al. 2017). Yet, specifying unimodal climatic responses through quadratic terms allows another way to fit this non‐linear interaction of energy and water availability. It is unclear exactly how to understand the complex shape of climatic effects on tree performance and trait selection, and these are probably not the best models for the job. But we do note that tall trees and those that have large seeds were more sensitive to the combination of hot and dry climates. Tall trees may be especially sensitive to this effect of heat and dryness as they need to pull water to great heights, resulting in very negative xylem tension. While the traits that confer rapid height growth and resultant great maximum height tend to trade‐off against hydraulic safety: large diameter vessels, low wood density, high sapwood conductivity and low P50 (Pfautsch et al. 2016; Towers et al. 2024; Portelli et al. 2023).

### 4.4 Caveats and Issues

#### 4.4.1 Sampling Bias and Resolution

Our analysis had to thread a path between the ecological processes, the covariates available and the plot data. The plot data were collected over decades from multiple campaigns with various objectives. The georeferencing was of variable quality with the oldest surveys predating Geographic Positioning Systems. Sampling was uneven over both geographic and environmental space, which can have detrimental effects on modelling. While our plots were mostly 30 m × 30 m, and we would like environmental data at that grain, this is not the case. Climate covariates were available at 30 arc sec, ~1 km cell‐side resolution, but still likely to show autocorrelation. Autocorrelation in residuals can result in reduced parameter uncertainty and may also lead to errors in estimates and reduced predictive performance. Yet methods to account for it can have unintended outcomes, most notably underestimating true environmental effects (Dormann et al. 2007).

#### 4.4.2 Intraspecific Trait Variation

Given attention to ITV, it is worth asking what effect its omission has. First, we repeat that our overarching use case is how to predict the responses of species across extensive landscapes from just a few traits. To this end, ITV, which undoubtedly occurs, is simply not accounted for in the model and its effects, if any, contribute to unmodelled variation. It is however important that trait sampling should ensure representative, unbiased, precise estimates of species means or medians (Albert et al. 2011). Second, ITV is typically smaller than between species variation especially over broader spatial scales (Siefert et al. 2015) and for some traits appears less strongly correlated with environment (wood density Fischer et al. 2026; SLA Dong et al. 2020). Third, how might ITV be accommodated in trait‐SDM? Peng et al. (2024) proposed a two‐step model to incorporate ITV‐environment relationships with a trait‐SDM. That yielded different relationships and predictions of occurrence. Such an approach demands further attention. Fourth, if ITV is aligned with that between species, along gradients with the same implications for environmental suitability and plant performance, we might expect that this would result in wider niches for each species. One result being that niche optima along the gradient will remain unchanged, thus having no effect on the trait‐environment term. Further though, this niche‐widening should apply for all species along the gradient. And so considering one species, the species either side of it along the gradient would also have their niches widened, which leads us to competition determining the presence and abundance of the species along the gradient in the manner we outlined above (Theory and response shape). So in summary, we see little impact of ignoring ITV in our models for inference. Considering ITV impacts on predictions requires greater attention to processes of assembly, which is beyond the scope of this paper, but an important avenue for future enquiry.

### 4.5 Single Versus Multi‐Trait Models

What do we make of single vs. multi‐trait models? or the purposes of inference, single trait models might represent maximum potential explanatory power of a trait, notwithstanding the possibility of dependences among traits. Interactions between traits are beyond the scope of this work. Multi‐trait models enable us to capture more of the richness of integrated phenotypes: representing strategies for facing the challenges of surviving and flourishing in their biotic and abiotic environments.

Future work characterising traits related to hydraulics and to roots would be beneficial. The root economic space could be informative with respect to resource supply, whether this would be better indicated by morphological traits such as root tissue density, root diameter of specific root length is an open question (Kou et al. 2026). In any case, isolating fine roots for species of trees in mixed forests would be challenging. Potentially arboretums or seedling studies offer easier initial steps. Hydraulic traits, while effortful, would be likely to be important to understanding further responses to water deficits (Pfautsch et al. 2016). Huber value (Sapwood area: Leaf Area ratio) is potentially a simple morphological trait of use here. Leaf petiole vasculature is a likewise a promising avenue to explore (Blackman et al. 2024).

Noting that this work is all observational, we point out that experimental studies planting beyond range margins may be helpful. Yet as we are concerned with realised niches, experiments would need to accommodate competition effects as well.

## 5 Conclusion

We have demonstrated that a few functional traits may provide useful predictors of environmental response, but not those that have necessarily been of focus previously (Table 1 and Westoby 1998; Pollock et al. 2012; Vesk et al. 2021). Commonly studied, and functionally important traits may not be selected in parsimonious multi‐trait and multi‐environment models of distributions. Stem sapwood density is a good predictor of environmental responses of eucalypt tree species over climatic gradients. Bark thickness deserves closer attention as it appears aligned to a growth‐defence trade‐off playing out over productivity gradients. These models offer prospects for predictions of species distributions from small numbers of traits. For planning and management, such trait‐SDMs can provide useful predictions of *where* certain kinds of species occur or could be restored into communities. But they will leave much uncertain, especially for identifying *which* particular species occur where. Lastly, we suggest that model testing is needed in different regions with different environments and subsets of species.

## Author Contributions

**Peter A. Vesk:** conceptualization (lead), formal analysis (lead), funding acquisition (equal), investigation (equal), methodology (equal), project administration (lead), resources (equal), supervision (equal), visualization (equal), writing – original draft (lead), writing – review and editing (equal). **Saras M. Windecker:** data curation (equal), investigation (equal), software (equal), writing – review and editing (equal). **Rachael V. Gallagher:** conceptualization (equal), writing – review and editing (equal). **William K. Morris:** data curation (equal), methodology (equal), resources (equal), visualization (equal), writing – review and editing (equal). **Laura J. Pollock:** investigation (equal), methodology (equal), resources (equal), writing – review and editing (equal). **Isaac R. Towers:** conceptualization (equal), writing – review and editing (equal). **Daniel S. Falster:** conceptualization (equal), funding acquisition (equal), supervision (equal), writing – review and editing (equal).

## Conflicts of Interest

The authors declare no conflicts of interest.

## Supporting information


**File S1:** ece373583‐sup‐0001‐Supplementarytable.xlsx.

## Figures and Tables

**FIGURE C1:**
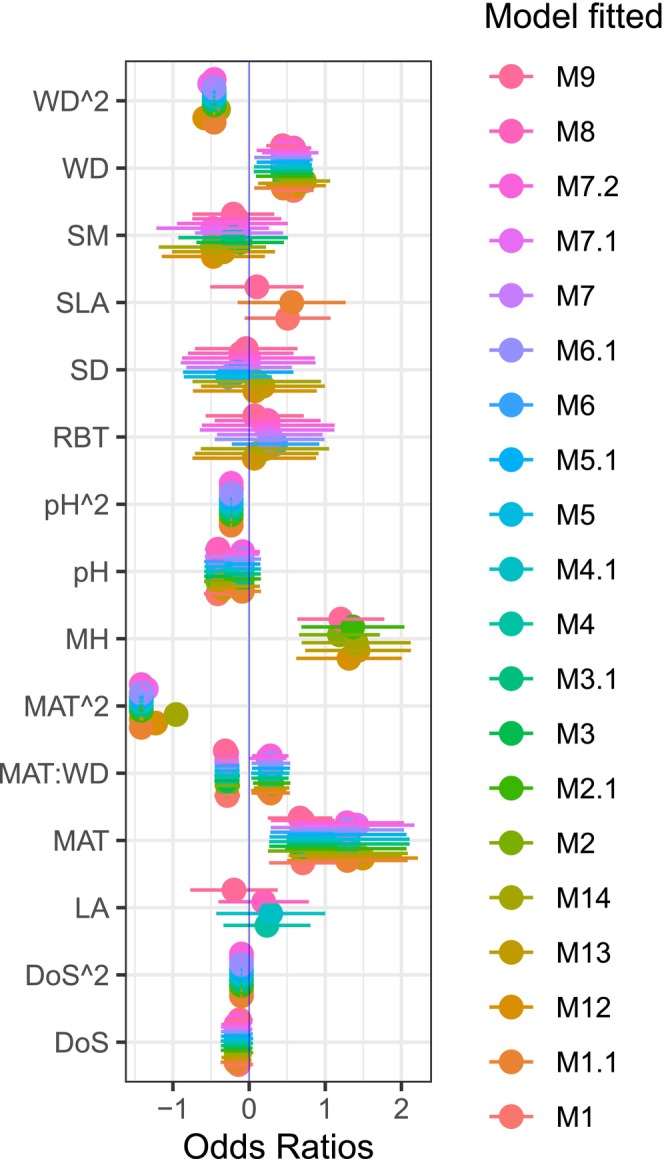
Plot of common effect terms from multiple models. These include models with linear environmental responses and single traits (M1, M2, M3, M4, M5, M6), models with linear environmental responses and multiple traits (M7, M8, M9), non‐linear (unimodal) environmental responses and single traits (M1.1, M2.1, M3.1, M4.1, M5.1, M6.1), non‐linear (unimodal) environmental responses and multiple traits (M7.1, M7.2, M12, M13, M14). Symbols are estimated coefficients and 90% CIs as bars.

## Data Availability

All data and code to produce the models and plots in this paper are available in Figshare: https://doi.org/10.26188/30715913.v1.

## Appendix A

**TABLE A1:** List of study taxa, ordered by frequency. Note, cryptic species and subspecies were combined in 17 cases. Taxa present in 10 or fewer grid cells were excluded.

Taxa	# grid cell presences
*Eucalyptus obliqua*	3764
*Eucalyptus cypellocarpa*	3579
*Eucalyptus radiata*	3527
*Eucalyptus sieberi*	3389
*Eucalyptus eugenioides & globoidea*	3152
*Eucalyptus viminalis* subsp. *viminalis & pryoriana*	2178
*Eucalyptus dives*	2092
*Eucalyptus pauciflora*	1959
*Eucalyptus macrorhyncha* subsp. *macrorhyncha*	1953
*Eucalyptus muelleriana*	1469
*Eucalyptus fastigata*	1352
*Eucalyptus dalrympleana* subsp. *dalrympleana*	1194
*Eucalyptus rubida* subsp. *rubida*	1159
*Eucalyptus polyanthemos*	1154
*Eucalyptus consideniana*	953
*Eucalyptus mannifera*	930
*Eucalyptus delegatensis* subsp. *delegatensis*	925
*Eucalyptus regnans*	877
*Eucalyptus melliodora*	873
*Eucalyptus bridgesiana*	813
*Angophora floribunda*	744
*Eucalyptus agglomerata*	730
*Corymbia gummifera*	707
*Eucalyptus elata*	681
*Eucalyptus globulus* subsp. *globulus*	667
*Eucalyptus yarraensis & ovata* subsp. *ovata*	636
*Eucalyptus baxteri*	597
*Eucalyptus botryoides*	504
*Eucalyptus croajingolensis*	496
*Corymbia maculata*	443
*Eucalyptus tricarpa* subsp. *tricarpa*	417
*Eucalyptus longifolia*	392
*Eucalyptus blakelyi*	390
*Eucalyptus rossii*	361
*Eucalyptus smithii*	358
*Eucalyptus cephalocarpa*	334
*Eucalyptus pilularis*	307
*Eucalyptus camphora* subsp. *humeana*	303
*Eucalyptus bosistoana*	298
*Eucalyptus camaldulensis* subsp. *camaldulensis*	290
*Eucalyptus tereticornis*	289
*Eucalyptus piperita*	279
*Eucalyptus paniculata*	271
*Eucalyptus albens*	230
*Eucalyptus angophoroides*	230
*Eucalyptus nitens*	210
*Eucalyptus stellulata*	200
*Eucalyptus fraxinoides*	198
*Syncarpia glomulifera* subsp. *glomulifera*	195
*Eucalyptus globulus* subsp. *maidenii*	172
*Eucalyptus baueriana*	157
*Eucalyptus saligna*	148
*Eucalyptus scias* subsp. *scias*	134
*Eucalyptus pauciflora* subsp. *debeuzevillei & niphophila & parvifructa*	127
*Tristaniopsis collina & laurina*	111
*Eucalyptus fibrosa* subsp. *fibrosa*	87
*Eucalyptus racemosa*	82
*Eucalyptus fulgens*	80
*Eucalyptus microcarpa*	75
*Eucalyptus punctata*	75
*Eucalyptus aromaphloia & sabulosa*	64
*Eucalyptus arenicola*	57
*Angophora costata* subsp. *costata*	52
*Eucalyptus chapmaniana*	52
*Eucalyptus falciformis*	52
*Eucalyptus cinerea*	45
*Eucalyptus leucoxylon* subsp. *leucoxylon & pruinosa*	44
*Eucalyptus burgessiana & stricta*	39
*Eucalyptus conspicua*	39
*Eucalyptus ignorabilis*	35
*Eucalyptus alaticaulis*	34
*Eucalyptus kybeanensis*	28
*Eucalyptus glaucescens*	27
*Eucalyptus amplifolia* subsp. *amplifolia*	26
*Eucalyptus globulus* subsp. *bicostata*	26
*Eucalyptus goniocalyx & nortonii & carolaniae*	24
*Eucalyptus sideroxylon* subsp. *sideroxylon*	22
*Eucalyptus quadrangulata*	20
*Eucalyptus willisii*	20
*Eucalyptus perriniana*	19
*Eucalyptus arenacea*	18
*Eucalyptus stenostoma*	18
*Eucalyptus serraensis*	17
*Eucalyptus tenella*	17
*Eucalyptus kitsoniana*	15
*Eucalyptus bunyip*	14
*Eucalyptus beyeriana*	13
*Eucalyptus strzeleckii*	13
*Eucalyptus alligatrix*	12
*Eucalyptus victoriana & verrucata*	12
*Corymbia eximia*	11
*Eucalyptus imitans*	11
*Eucalyptus mackintii*	11
*Eucalyptus viminalis* subsp. *cygnetensis*	11

## Appendix B

**TABLE B1:** Summary statistics of traits for 94 eucalypt taxa with woody species from AusTraits 5.0, in brackets (Falster et al. 2021). (AusTraits 5.0 accessed 16 Oct 2023).

Quantile	SLA (mm^2^ mg^−1^)	LA (cm^2^)	SM (mg)	Height (m)	SD (g mL^−1^)	Bark thickness (mm)^a^
0%	2.7 (0.7)	8 (0.01)	0.16 (0.01)	4 (0.01)	0.53 (0.15)	1.7 (0.1)
1%	2.9 (1.5)	8.5 (0.02)	0.21 (0.04)	4.9 (0.04)	0.53 (0.28)	2.6 (0.2)
5%	3.4 (2.2)	11.4 (0.09)	0.23 (0.12)	7.7 (0.3)	0.54 (0.38)	6.2 (0.4)
10%	3.6 (2.6)	12.1 (0.18)	0.31 (0.28)	10 (0.4)	0.56 (0.44)	10.1 (0.5)
25%	4.1 (3.8)	17.5 (1.18)	0.45 (1.08)	20 (1.08)	0.58 (0.54)	15.9 (0.7)
50%	4.9 (6.1)	21.3 (10.7)	0.64 (4.32)	30 (1.8)	0.60 (0.64)	20.3 (1.3)
75%	5.5 (10.1)	29.4 (29.3)	1.17 (16.4)	40 (5.5)	0.63 (0.74)	26.2 (4.5)
90%	6.4 (14.6)	35.1 (60.0)	1.93 (63.9)	58.5 (15.5)	0.72 (0.86)	32.1 (9.3)
95%	7.1 (14.6)	41.3 (105)	3.02 (201)	70 (25)	0.79 (0.93)	33.5 (11.4)
99%	8.6 (19.3)	57.9 (314)	8.03 (3600)	90 (38.5)	0.83 (1.01)	36.9 (17.7)
100%	9.8 (52.3)	98.7 (2400)	10.3 (8333)	90 (85.4)	0.87 (1.23)	44 (24.7)

^a^ AusTraits bark thickness is raw measurement, whereas our Relative Bark Thickness data for this paper are standardised for a 30 cm girth stem.

## Appendix C

### Sensitivity to Model Selection

In this Appendix, we analyse the sensitivity of the inferences to model specification. We first examine the common effects (response of the hypothetical average eucalypt with average traits). We then examine the more relevant trait‐environment terms. We varied single vs. multi‐trait models and linear vs. unimodal (quadratic) environmental responses.

### Common Effects, Comparing Linear and Quadratic Environmental Responses

Broadly, the common effects were consistent across single trait models with either linear or quadratic responses to the environment (Figure C1). Two types of exceptions were that, first, linear effects of environment increased in magnitude when quadratic terms were included. The quadratic terms were uniformly negative, indicating unimodal environmental niches. So, the quadratic term allows the linear term to increase in magnitude, without predicting endless increase along the gradients.

A second class of exceptions concerned the MAT:WD interaction, which changed from negative in linear response models to positive in quadratic response models (Figure C1). This appears to offset the predicted low likelihood of occurrence of eucalypts at hot and dry sites when quadratic terms are included. That is, the shape of the average eucalypt response to joint distributions of climate is complex.

In single trait models, common environment effects were very similar to multi‐trait models (Figure C1).

### How Did Quadratic Terms for Unimodal Species Responses to Environment Affect Trait‐Environment Terms?

We compared the trait‐environment terms from the six single‐trait models, with and without quadratic terms for the environmental covariates MAT, WD, pH, DoS (Figure C2). The coefficients were similar in sign and magnitude regardless of inclusion of quadratic terms for environmental responses. The confidence intervals for the respective terms overlap greatly, and nearly always overlap the point estimate of the contrasted model. Thus, there is no support for the hypothesis that inferences on trait‐environment terms from models assuming linear species response are different from those assuming quadratic (unimodal) responses. There was a trend for the quadratic models to yield estimates that were less certain than those assuming linear responses.

### Trait‐Environment Effects in Single‐Trait and Multi‐Trait Models with Unimodal Environmental Responses

Trait‐Environment terms were generally similar, though sometimes stronger and more certain, in single‐trait than in multi‐trait models (Figure C3). Coefficients were similar within the margin of error; confidence intervals overlapped the point estimates of the contrasting model. For some trait‐environment terms, an effect observed in the single trait model became negligible in the multi‐trait model. Yet, even in single trait models, SLA, LA and maximum height explained relatively little between‐species variance in environmental responses compared to stem sapwood density and/or bark thickness (Figure 7). Here we note differences from the multi‐traits model we focus on for most of the paper.

In single‐trait quadratic models, SLA and MH had significant Trait‐Environment terms (Figure C3). This indicated that high SLA taxa were more likely to increase with Temperature. Higher SLA increased the occurrence, but only in a single trait model.

A model with only the Maximum Height trait indicated stronger effects on climate responses with more positive response to MAT (Figure C3). Taxa with greater Maximum Height decreased with Water Deficit. Maximum height positively influenced the occurrence of tree species, in both single and multi‐trait models. However, that effect of maximum height in multi‐trait models was subsumed by that of SD or BT models, which were both weakly correlated with MH (*r* = 0.19, 0.2, respectively). Taller species had lower density wood and had thicker bark. As noted by Portelli et al. (2023) there is some possible confounding in that bigger trees often have a skirt of thick, rough bark extending above breast height where thickness is measured.

Leaf Area had no significant trait‐environment effects but trended to bigger leaved species being more common in wetter environments.

Both Stem Sapwood Density and Bark Thickness had significant Trait‐Environment terms for all three of MAT, WD and the MAT:WD interaction in single trait models. These terms were altered in a mult‐trait model (M13). For instance, a negative term for SD and MAT (M5.1). Possibly this was explained by RBT explaining response to temperature and SD and RBT being negatively correlated. Conversely, RBT had a negative effect on water deficit response in the single trait model (M6.1), but this diminished in the multi‐trait model, where the WD response was influenced by stem sapwood density.

Another example of changed coefficients is MH:MAT; a positive effect of taller maximum height on temperature response observed in the single trait model (M2.1) became smaller and non‐significant in the multi‐trait model (M13).

## Appendix D

**TABLE D1:** Predicted niche optima using the chosen model, M13. For mean values of the trait and ±2 standard deviations. All trait data were log transformed before calculation.

Environment	Trait	Low trait value (−2 SD)	Mean trait value	High trait value (+2SD)
MAT (°C)	SD	13.7	13.4	13.1
MAT (°C)	RBT	11.2	13.4	15.5
MAT (°C)	SM	12.9	13.4	13.8
MAT (°C)	MH	12.6	13.4	14.2
WD (mm year^−1^)	SD	254	473	691
WD (mm year^−1^)	RBT	462	473	483
WD (mm year^−1^)	SM	470	473	475
WD (mm year^−1^)	MH	548	473	397
DoS (m)	SD	0.857	0.844	0.832
DoS (m)	RBT	1.11	0.844	0.579
DoS (m)	SM	1.12	0.844	0.565
DoS (m)	MH	0.844	0.844	0.845
pH	SD	4.02	4.44	4.87
pH	RBT	4.28	4.44	4.61
pH	SM	4.62	4.44	4.27
pH	MH	4.46	4.44	4.42
